# Emergency ambulance service involvement with residential care homes in the support of older people with dementia: an observational study

**DOI:** 10.1186/1471-2318-14-95

**Published:** 2014-08-28

**Authors:** Sarah Amador, Claire Goodman, Derek King, Ina Machen, Natasha Elmore, Elspeth Mathie, Steve Iliffe

**Affiliations:** 1 Centre for Research in Primary and Community Care, University of Hertfordshire, Hatfield AL109AB, UK; 2 Personal Social Services Research Unit, Cowdray House, The London School of Economics and Political Science, Houghton Street, London, WC2A 2AE, UK; 3 Primary Care Unit, Institute of Public Health, Forvie Site, University of Cambridge, Robinson Way, Cambridge, CB2 0SR, UK; 4 Department of Primary Care and Population Sciences, University College London, London, NW32PF, UK

**Keywords:** Care homes, Aged, Health services for the aged, Dementia, Emergency medical services, Longitudinal

## Abstract

**Background:**

Older people resident in care homes have a limited life expectancy and approximately two-thirds have limited mental capacity. Despite initiatives to reduce unplanned hospital admissions for this population, little is known about the involvement of emergency services in supporting residents in these settings.

**Methods:**

This paper reports on a longitudinal study that tracked the involvement of emergency ambulance personnel in the support of older people with dementia, resident in care homes with no on-site nursing providing personal care only. 133 residents with dementia across 6 care homes in the East of England were tracked for a year. The paper examines the frequency and reasons for emergency ambulance call-outs, outcomes and factors associated with emergency ambulance service use.

**Results:**

56% of residents used ambulance services. Less than half (43%) of all call-outs resulted in an unscheduled admission to hospital. In addition to trauma following a following a fall in the home, results suggest that at least a reasonable proportion of ambulance contacts are for ambulatory care sensitive conditions. An emergency ambulance is not likely to be called for older rather than younger residents or for women more than men. Length of residence does not influence use of emergency ambulance services among older people with dementia. Contact with primary care services and admission route into the care home were both significantly associated with emergency ambulance service use. The odds of using emergency ambulance services for residents admitted from a relative’s home were 90% lower than the odds of using emergency ambulance services for residents admitted from their own home.

**Conclusions:**

Emergency service involvement with this vulnerable population merits further examination. Future research on emergency ambulance service use by older people with dementia in care homes, should account for important contextual factors, namely, presence or absence of on-site nursing, GP involvement, and access to residents’ family, alongside resident health characteristics.

## Background

UK studies on the use by care homes of emergency ambulance services are incidental to wider studies investigating the impact of care home residents on hospital emergency departments, and based on either prospective [1,2] or retrospective analysis of Hospital Episode Statistics (HES) [3-5]. Despite evidence of a relationship between hospital emergency admissions and levels of nursing care [6], studies do not discriminate between care homes with and without on-site nursing provision. Older people resident in care homes have a limited life expectancy [7], in the UK about two-thirds have dementia [8]. It is estimated that one third of people with dementia are living in a care home [8]. Nearly 12,500 social care providers registered with the Care Quality Commission (i.e. the independent regulator of all health and social care services in England) to provide regulated services, operate in just over 25,000 locations in England. Just over half of these locations are residential care homes, that is, care homes with no on-site nursing that provide personal care only [9], which are entitled to the full range of NHS primary, community and hospital-based care support.

A target of end-of-life care programmes and interventions in care homes is to reduce unplanned admissions to hospital. Studies on admissions from care homes have found that up to 95% of residents were transferred from the care home to hospital via emergency ambulance [4] yet little is known about the involvement of emergency ambulance services in the UK. Several international studies have reported important variations in ED use between nursing homes [10-12] significantly associated with factors which include gender [10], length of stay in the care home [13], differential access to medical care for common conditions (e.g. pneumonia) [14], and how the care home is funded [12].

This paper considers the findings from a longitudinal study of people with dementia resident in six UK residential care homes. It focuses on the use of emergency ambulance services in particular. We examine the frequency, reasons, outcomes and factors associated with emergency ambulance service use. Specifically, this paper asks what characteristics of residents are associated with emergency ambulance service use. We predicted that emergency ambulance use is related to case complexity as measured by number of co-morbidities and use of other services, as well as age.

## Methods

Care homes were identified from the Care Quality Commission (previously known as the Commission for Social Care Inspection) directory of care homes using the following criteria: the care home is (i) for older people, offering personal care and specialist support in dementia care, (ii) does not have onsite nursing care, (iii) has on average between 20 and 50 places, (iii) the most recent CQC inspection report is favourable with no on-going problems or issues requiring action, (iv) the care homes’ typicality is comparable with findings from other national studies and similar to one another, (v) care home staff consider they have a good working relationship with their local primary care services and (vi) the final sample has a mix of ownership and geographical location. Inclusion criteria for care home residents were that they were 65 years or older, with a documented diagnosis of dementia, or assessment by the senior care worker that the older person had cognitive impairment indicative of dementia and a validated measure of cognitive function impairment. Exclusion criteria included individuals who lacked capacity to consent and for whom a consultee could not be identified. Further details regarding the recruitment process used for both care homes and older people with dementia are published elsewhere [15].

The study prospectively tracked the events and care experienced by older people with dementia living in six residential care homes located in the East of England over 12 months beginning March 2009.

Data were extracted from the residents’ care home notes at four-monthly intervals to establish services received and care provided. Services received were extracted using a modified version of the Client Service Receipt Inventory (CSRI) [16]. Care note data of residents who died were complemented by interviews with care home staff. Care home characteristics were collected using Annual Quality Assurance Assessment (AQAA) forms completed for the research team by care home managers. Missing data were obtained through Care Quality Commission (CQC) listings and interviews with the care home manager.

Emergency ambulance service use was defined as resident contact with an emergency ambulance or not. The main reason for each emergency ambulance contact recorded over the period of data collection was extracted from the care notes and classified by main reason for contact. Main reasons for contacts were then further broken down according to outcome, that is, according to whether contact resulted in (i) non-conveyance (i.e. the resident was attended to by emergency practitioners at the home and not conveyed to hospital) (ii) same day discharge (i.e. the resident was conveyed to hospital A&E and then discharged back to the care home with no overnight stay in a hospital ward), or (iii) unscheduled admission.

The logistic regression analyses adjusted for factors potentially associated with emergency ambulance service use. Based on empirical findings on emergency department use by long-term care residents as reviewed above, the adjustment variables selected were the age of the resident, gender, length of residency in the care home, number of co-morbidities, admission route into the care home, use of other services (i.e. general practitioner, district nurse) and the care home itself. All covariates were entered into the model. The dependent variable is contact with an emergency ambulance or not. Clustered standard errors were used to account for the potential effect of the care home on use of an emergency ambulance. We predicted that emergency ambulance use is related to case complexity as measured by number of co-morbidities and contacts with general practitioners and district nurses, as well as age. The goodness of fit of the logistic regression model was assessed by firstly, comparing the full model with a constant only model to determine the level of significance of the set of independent variables; and further by assessing the Nagelkerke’s R-squared statistic [17], the percentage of observations in which the model correctly predicted the dependent variable, the Link test and the Hosmer-Lemeshow test. Data was analysed using Stata 10.1 [18].

This study (REC reference: 08/H0502/74) received a favourable ethical opinion from the Southampton & South West Hampshire Research Ethics Committee (A) on 14 July 2008. Informed consent was elicited from residents and/or consultees for the review of care home notes. Informed consent was elicited from interview participants.

## Results

Ten care homes were identified following inclusion criteria detailed above and of these six agreed to participate. A total of 214 residents across the six care homes were eligible to participate in the study. Of these, 133 residents (62.1%) were recruited in total. Residents who did not participate in the study were those who lacked capacity to consent and whose personal consultees either did not think the study would be of interest to the resident or did not respond despite letter and telephone follow-up.

### Characteristics of the care homes

Care homes that participated were a mix of provider type, size, location, building structure and religious affiliation (Table 1). All were residential homes, that is, long-term care facilities without on-site nursing that provide personal care only. Care homes (CHs) had between 46 and 67 places.

**Table 1:** Care home characteristics*

**Care Home**	**CH1**	**CH2**	**CH3**	**CH4**	**CH5**	**CH6**
Provider type	Private not for profit	Private not for profit	Private not for profit	Voluntary	Private	Private
Number of places	46	62	60**	66	67	57
Number of dementia places	46	61	60	66	67	57
Location	Suburban	Urban	Suburban	Urban	Rural	Rural
Building	Local authority	Purpose built	Purpose built	Purpose built	Conversion	Purpose built
Religious affiliation	No	No	No	Yes	No	No

*Source: CQC listings AQAA data and Manager Interviews.

**1 place learning disability.

### Baseline characteristics and service use

The mean age of residents was 86.2 (±SD 6.9) years. Those recruited were exclusively white and mostly female (n = 103; 77.4%). The mean length of stay was 2.2 years (±SD 1.9). The mean number of co-morbidities was 2.4 (±SD 1.5). In addition to dementia, 44% (n = 58) of residents were recorded in their care home notes as having three or more co-morbidities, the most commonly recorded physical condition being heart disease (26% of the sample). 43% of residents were admitted to the care home from their own home, 6% were admitted from a relative’s home, 29% from hospital, 11% from another care home and 11% from sheltered/warden controlled housing. Sheltered accommodation refers to housing aimed at people 55 years and over, which offer independent living with extra assistance if needed. These are run by managers or wardens who work office hours, and who provide suitable support to the resident (e.g. repairs, emergencies). Residents also have access to 24-hour emergency care assistance via an alarm system linked to a monitoring centre.

The most frequently used services by residents were: hospital services including emergency ambulance services, emergency department services, inpatient and outpatient services; community health services included district nursing (DN) as well as Out-of-Hours General Practitioner (OOHs GP) services; primary health services including general practitioner (GP) services. Median number of contacts with primary care services per month was 0.9 (IQR = 0.5-1.3). Median number of contacts with community health services, hospital services and emergency ambulance services specifically was 0.4 (IQR = 0.2-1.1), 0.3 (IQR = 0–0.8) and 0.1 (IQR = 0–0.3) respectively.

### Main reasons for emergency ambulance call outs and outcomes

Older people with dementia come into contact with emergency ambulances for clinical events (see Table 2) the most common being trauma following a fall in the home (42% of all emergency ambulance contacts). Other reasons for contacts with an emergency ambulance as recorded in the care notes include respiratory problems (9% of all emergency ambulance contacts), cardiovascular complaints (7%), gastrointestinal complaints (6%) and genitourinary complaints (6%).

**Table 2:** Main reasons and outcomes of emergency ambulance call outs to care homes

**Reason for emergency ambulance call out as recorded in care notes**	**Outcomes as recorded in care notes**				
	**Non**-conveyance*	**Same**-day discharge**	Unscheduled admittance***	**Total N**	**Total%**
Trauma^†^	13	23	24	60	41.7
Respiratory	1	4	8	13	9.0
Cardiovascular Complaint	0	3	7	10	6.9
Gastrointestinal Complaint	0	1	7	8	5.6
Genitourinary Complaint	0	8	0	8	5.6
Altered Mental Status	0	3	4	7	4.9
Non-specific Complaint	2	2	2	6	4.2
Cerebrovascular Complaint	0	1	4	5	3.5
Muscoskeletal No Trauma	0	2	2	4	2.8
Seizure	0	0	3	3	2.1
Circulatory Complaint	0	0	1	1	0.7
Ear, Nose & Throat Problem	1	0	0	1	0.7
Unknown (Missing^‡^)	18	0	0	18	12.5
Total N	35	47	62	144 (100%)	
Total%	24.3	32.6	43.1		

*Resident attended to by emergency practitioner at the care home; not conveyed to hospital.

**Resident conveyed to hospital A&E; discharged back to the care home on the same day with no overnight stay in hospital ward.

***Resident conveyed to hospital A&E; admitted to hospital ward for overnight stay.

^†^Only 3 out of 60 emergency ambulance call outs for trauma were unrelated to falls in the home.

^‡^The CSRI captures reasons for admission to A & E and hospital, but not reasons for emergency ambulance call outs when there is no conveyance to either. As such, for 18 call outs resulting in non-conveyance, reasons were not extracted from care notes and remain unknown. However, the majority are likely to be due to trauma following a fall in the home.

Emergency ambulance contacts resulted in admission to hospital in less than half of all cases (40% of all trauma related contacts specifically). A third of contacts resulted in same day discharges, that is, residents being returned to the home following assessment in A&E (38% of all trauma related contacts specifically). A quarter of all contacts resulted in non-conveyance following assessment by emergency staff in the home (22% of trauma related contacts specifically).

### Impact of resident characteristics and care homes on contact with emergency services

Logistic regression models assessed if contact with emergency ambulance services was associated with resident characteristics. Table 3 gives the unadjusted and adjusted odds ratios and 95 per cent confidence intervals of contact with emergency services by resident characteristics. In the unadjusted models, there is a 6.1% and a 15% increase in the odds of contact with emergency ambulance services for each additional year of age and each contact with a general practitioner respectively. The effects of gender, length of residency, number of co-morbidities, admission route into the home and number of contacts with a district nurse were not larger than could be due to chance in these data (p > 0.05).

**Table 3:** **Unadjusted and adjusted odds ratios** (**OR**) **and 95**% **confidence intervals of contact with emergency ambulance services**

**Variable**	**Unadjusted OR (95%CI)**	**P**-**value**	**Adjusted OR (95%CI)**	**P**-**value**
**Age**	1.061 (1.006, 1.118)	0.028	1.067 (0.997, 1.142)	0.060
**Gender**				
Male	1.00		1.00	
Female	0.551 (0.235, 1.292)	0.170	0.661 (0.385, 1.135)	0.133
**Length of residency**	1.001 (0.837, 1.197)	0.989	0.965 (0.735, 1.266)	0.796
**Number of co**-**morbidities**	1.204 (0.944, 1.536)	0.134	1.207 (0.879, 1.655)	0.245
**Admission route**		0.174		
Own home	1.00		1.00	
Relative’s home	0.104 (0.012, 0.930)	0.043	0.092 (0.017, 0.493)	0.005
Hospital	0.590 (0.248, 1.405)	0.233	0.556 (0.136, 2.283)	0.416
Other care home	0.391 (0.112, 1.362)	0.140	0.284 (0.063, 1.277)	0.101
Sheltered housing/Warden controlled	1.000 (0.287, 3.488)	1.000	1.055 (0.349, 3.186)	0.925
**Number of General Practitioner contacts**	1.149 (1.052, 1.253)	0.002	1.170 (1.012, 1.351)	0.034
**Number of District Nurse contacts**	1.048 (0.978, 1.124)	0.184	1.003 (0.926, 1.086)	0.943
	Link test p-value (prob _hatsq)			0.232
	Hosmer-Lemeshow (groups = 10) chi2 (8)			0.1947

A test of the full model against a constant only model was statistically significant, indicating that predictors as a set, effectively distinguished between residents who had contact with emergency services and those who did not (chi square = 40.037, p < 0.001 with df = 15). Nagelkerke’s R2 of .379 indicated a relatively good relationship between prediction and grouping. Prediction success overall was 80% (75.4% for no contact and 84.1 for contact with emergency services). The Link test and Hosmer-Lemeshow test p-values were not significant, supporting the model specification chosen.

In the adjusted model, the coefficient for the variable ‘number of contacts with a general practitioner’ indicates that when contacts with a general practitioner is increased by one unit, residents had 1.235 times greater odds (i.e. 17% increase in the odds) of using emergency ambulance services. The odds of using emergency ambulance services for residents admitted from a relative’s home were 90% lower than the odds of using emergency ambulance services for residents admitted from their own home. This is irrespective of residents’ age, gender, length of residency in the care home, number of co-morbidities, number of contacts with general practitioners and number of contacts with district nurses.

## Discussion

The most common reason for contact with an emergency ambulance was trauma following a fall in the home, resulting in a quarter of all cases in non-conveyance, and in a third of all cases in same-day discharge back to the care home following assessment at the ED. The large proportion of emergency ambulance contacts that do not result in unscheduled admittance to hospital, suggest that at least a reasonable proportion of these is for ambulatory care sensitive conditions (ACSC), that is, physical health conditions such as pneumonia and gastroenteritis that can potentially be treated safely in a care home [19]. Results also show that emergency ambulance service use is positively associated with general practitioner contacts, but not district nurse contacts or number of co-morbidities. Together, these results suggest that in residential care homes with no on-site nursing, patients with acute health needs (possibly including ACSCs) are likely to require both primary care and ambulance services. Indeed, patients and homes who frequently call on GPs may be more likely to find that on a given occasion, a GP cannot meet their needs and therefore call on an ambulance instead.

An emergency ambulance is not likely to be called for older rather than younger residents (although there is a trend), nor for women more than men. Length of residence does not influence use of emergency ambulance services. These results would appear to lend credence to those from a recent study examining the effect of severity of cognitive impairment on ED use by nursing home residents [20] that show odds of ED utilisation to decrease with more advanced dementia, potentially reflecting differential treatment patterns and family preferences regarding intensity of treatment (i.e. palliative rather than more aggressive approaches) at different levels of cognitive impairment.

Finally, results suggest that for older people with dementia, other factors beyond residents’ health are associated with the use of emergency ambulance services. Specifically, for those residents with dementia who were admitted from relatives’ homes, it is possible that care home staff were better informed and more confident in their assessment of the need for emergency transfer, owing to greater involvement of relatives, with more intimate knowledge of the resident.

To our knowledge, this is the first UK-based study to report on the involvement of emergency ambulance services with care homes that do not have on-site nursing provision and with older people with dementia resident in care homes in particular. An important methodological shortcoming of the study is that necessary limitations on sample size and diversity do not ensure generalisability of the data. Whilst the sample of care homes and residents generates useful insights, it cannot be assumed that the findings from these homes, in this region, could be applied to all UK settings. Care home notes from which resident baseline characteristics were extracted could be inconsistent across timepoints. Residents’ long-term conditions are likely to be underreported as a result. The relatively small sample size is acknowledged as a limitation inherent to the longitudinal design of the study. Measures employed for data collection (resulting in missing data as regards reasons for emergency ambulance call outs) are acknowledged as a limitation characteristic of studies in this area.

Care homes that do not have clinically qualified staff rely on primary care and emergency services for medical and nursing support at times of crisis. The number of contacts that did not require a hospital admission suggests that there is scope to dramatically reduce the demand on emergency services. Our results fit with the emergency department literature reviewed here that demonstrate care homes’ demand on emergency services to be highly variable, and linked to multiple causes. Reasons for calling an emergency ambulance as documented by care workers in this study, mirror chief complaints as documented by hospital based clinicians reported elsewhere [3]. The paucity of information surrounding emergency call outs to care homes that result in non-conveyance to hospital is due in part to the widespread reliance on HES data highlighted above. Refinement of data collection methods in future research on emergency service involvement with care homes is needed to fully address decision-making in relation to outcomes (i.e. whether residents are treated on-site or conveyed to hospital) and understand potentially emerging issues surrounding non-conveyance of older people with dementia resident in care homes.

## Conclusion

Emergency ambulance service use by older people with dementia in residential care homes is high, associated with ambulatory care sensitive conditions and to some extent predictable. Future research should account for important contextual factors, namely, presence or absence of on-site nursing, GP involvement, and access to residents’ family, alongside resident health characteristics.

## Abbreviations

ED: Emergency department; CH: Care home; HES: Hospital episode statistics.

## Competing interests

The authors declare that they have no competing interests.

## Authors' contributions

CG conceived the study, made substantial contributions to the analysis and interpretation of data and the drafting of the manuscript. SA made substantial contributions to the acquisition, analysis and interpretation of data, and the drafting of the manuscript. DK made substantial contributions to the analysis and interpretation of data. NE, IM and EM made substantial contributions to the acquisition, analysis and interpretation of data. SI was involved in revising the manuscript critically for important intellectual content. All authors have given final approval of the version to be published.

## Pre-publication history

The pre-publication history for this paper can be accessed here:

http://www.biomedcentral.com/1471-2318/14/95/prepub
